# ﻿A new species of *Cyrtodactylus* Gray, 1827 (Squamata, Gekkonidae) from southwestern Yunnan, China

**DOI:** 10.3897/zookeys.1084.72868

**Published:** 2022-01-27

**Authors:** Shuo Liu, Dingqi Rao

**Affiliations:** 1 Kunming Natural History Museum of Zoology, Kunming Institute of Zoology, Chinese Academy of Sciences, 32 Jiaochang Donglu, Kunming, Yunnan 650223, China Kunming Institute of Zoology, Chinese Academy of Sciences Kunming China; 2 Kunming Institute of Zoology, Chinese Academy of Sciences, No.17 Longxin Road, Kunming, Yunnan, 650201, China Kunming Institute of Zoology, Chinese Academy of Sciences Kunming China

**Keywords:** Bent-toed gecko, *Cyrtodactyluschauquangensis* group, Menglian County, taxonomy

## Abstract

A new species of the *Cyrtodactyluschauquangensis* species group is described based on four specimens collected from the karst formations of Menglian County, Puer City, Yunnan Province, China. The new species can be separated from all other congeners by having a unique combination of morphological characters: a medium-sized body; ventrolateral folds present with interspersed small tubercles; seven precloacal pores in a continuous series in males, absent in females; enlarged femoral scales and femoral pores absent; two postcloacal tubercles on each side; and one or two rows of enlarged subcaudals. Genetically, the new species most closely related to *C.wayakonei* and the uncorrected sequence divergences of the ND2 gene and its flanking tRNAs between the new species and investigated congeners range from 7.2% to 18.4%.

## ﻿Introduction

*Cyrtodactylus* Gray, 1827 is the most speciose and ecologically diverse gekkotan genus with more than 300 recognized species so far (Grismer et al. 2021a, b; Uetz et al. 2021). Grismer et al. (2021a) partitioned the species of *Cyrtodactylus* into 10 ecotypes according to their habitat preferences. Of the 10 ecotypes, the karst ecotype is the second largest and contains the majority of the most recently described species (Grismer et al. 2021a).

The *Cyrtodactyluschauquangensis* Hoang, Orlov, Ananjeva, Johns, Hoang & Dau, 2007 species group, previously *C.wayakonei* Nguyen, Kingsada, Rösler, Auer & Ziegler, 2010 species group, is a karst ecotype species group, which is distributed in northern Indochina, ranging from northern Thailand and Laos to northwestern and central Vietnam, and to Yunnan Province in southern China (Grismer et al. 2021a, b). This species group contains 23 named species to date (Liu and Rao 2021a; Liu et al. 2021; Zhang et al. 2021).

During our fieldwork in southern Yunnan Province, China, in 2021, some specimens of *Cyrtodactylus* were collected from the karst formations of Menglian County, Puer City. Morphological and molecular phylogenetic analyses revealed that the new collection belonged to an unnamed species of the *C.chauquangensis* species group. We herein describe it as a new species.

## ﻿Materials and methods

### ﻿Sampling

Specimens were collected by hand. Photographs were taken to document the color pattern of specimens in life prior to their euthanization. Liver tissues were stored in 99% ethanol and specimens were preserved in 75% ethanol. Specimens were deposited at Kunming Natural History Museum of Zoology, Kunming Institute of Zoology, Chinese Academy of Sciences (**KIZ**).

### ﻿Morphological analyses

Measurements were taken with digital calipers to the nearest 0.1 mm. Bilateral scale counts were given as left/right. The methodology of measurements and meristic counts followed Liu and Rao (2021a, b):

**AG** axilla to groin distance;

**DTR** dorsal tubercle rows, number of dorsal, longitudinal rows of tubercles at midbody between ventrolateral folds;

**ED** ear diameter, greatest diameter of ear;

**EE** eye orbit to ear distance, from posterior corner of eye orbit to anterior margin of ear opening;

**EFS** enlarged femoral scales, number of enlarged femoral scale beneath each thigh;

**ForeaL** forearm length, from base of palm to elbow;

**FP** femoral pores;

**GSDT** granular scales surrounding dorsal midbody tubercles;

**HH** maximum head height, from occiput to underside of jaws;

**HL** head length, from tip of snout to posterior margin of retroarticular of lower jaw;

**HW** maximum head width;

**I** postrostrals or internasals;

**IFL** infralabials;

**IND** internarial distance, measured between inner borders of nostrils;

**IOD** interorbital distance, measured across narrowest point of frontal bone;

**LF4** subdigital lamellae under the fourth finger;

**LT4** subdigital lamellae under the fourth toe;

**ML** mental length;

**MW** mental width;

**OD** greatest diameter of orbit;

**PAT** postcloacal tubercles, number of tubercles on each side of postcloacal region;

**PM** postmentals, scales bordering mental shield, except infralabials;

**PP** precloacal pores;

**PVT** paravertebral tubercles, counted in a single paravertebral row from the level of the forelimb insertions to the level of the hind limb insertion;

**RH** rostral height;

**RW** rostral width;

**SE** snout to eye distance, from tip of snout to anterior corner of eye orbit;

**SL** shank length, from the base of heel to the knee;

**SPL** supralabials;

**SVL** snout–vent length, from tip of snout to anterior margin of cloaca;

**TaL** tail length, from posterior margin of cloaca to tip of tail;

**V** longitudinal ventral scale rows, counted across the belly between the ventro­lateral folds at midbody.

Morphological comparisons were based on the original descriptions of each species (Hoang et al. 2007; Bauer et al. 2009, 2010; Ngo and Grismer 2010; Nguyen et al. 2010, 2015b, 2017; Sumontha et al. 2010; Luu et al. 2011; Ngo 2011; Ngo and Chan 2011; Kunya et al. 2014; Nazarov et al. 2014; Nguyen et al. 2014; Schneider et al. 2014, 2020; Le 2016; Pham et al. 2019; Liu and Rao 2021a; Liu et al. 2021; Zhang et al. 2021).

### ﻿Molecular analyses

Molecular data were generated for three specimens collected from Menglian County, Puer City, Yunnan Province, China, and available sequences of the *Cyrtodactyluschauquangensis* species group were obtained from GenBank; the new sequences have been deposited on GenBank under the accessions OM296042–OM296044. *Cyrtodactylusdattkyaikensis* Grismer, Wood, Quah, Grismer, Thura, Oaks & Lin, 2020 and *C.sinyineensis* Grismer, Wood Jr, Thura, Zin, Quah, Murdoch, Grismer, Lin, Kyaw & Lwin, 2017 were used as the outgroups according to Liu et al. (2021). Total genomic DNA was extracted from liver tissue stored in 99% ethanol using a DNeasy blood and tissue kit, Qiagen (California, USA). A fragment of the NADH dehydrogenase subunit 2 (ND2) gene and its flanking tRNAs was amplified and sequenced using the primers L4437b and H5934 (Macey et al. 1997). The experiment protocols used in this study are the same as Liu et al. (2021). Sequences were edited and assembled using SeqMan in Lasergene 7.1 (DNASTAR Inc., Madison, WI, USA) and MEGA X (Kumar et al. 2018).

Sequences were aligned using ClustalW (Thompson et al. 1994) with default parameters. The best-fit substitution models were chosen using the Bayesian Information Criterion (BIC) in ModelFinder (Kalyaanamoorthy et al. 2017) for IQ-TREE and MrBayes, respectively. Maximum likelihood analysis was performed in IQ-TREE 1.6.12 (Nguyen et al. 2015a) used the TIM+F+I+G4 model for the first codon position, the second codon position, and the tRNAs; and the GTR+F+R2 model for the third codon position. One thousand bootstrap pseudoreplicates via the ultrafast bootstrap approximation algorithm were used to construct a final consensus tree. Nodes with ultrafast bootstrap values of 95 and above were considered significantly supported (Minh et al. 2013). Bayesian inference was performed in MrBayes 3.2.6 (Ronquist et al. 2012) used the GTR+F+I+G4 model of evolution for the first codon position, the second codon position, and the tRNAs; and GTR+F+G4 model for the third codon position. The chains were run for 1,000,000 generations and sampled every 100 generations. The first 25% of the sampled trees was discarded as burn-in and then the remaining trees were used to estimate Bayesian posterior probabilities. Nodes with Bayesian posterior probabilities of 0.95 or higher were considered well-supported (Huelsenbeck et al. 2001; Wilcox et al. 2002). Pairwise distances between species were calculated in MEGA X (Kumar et al. 2018).

## ﻿Results

**Figure 1.**
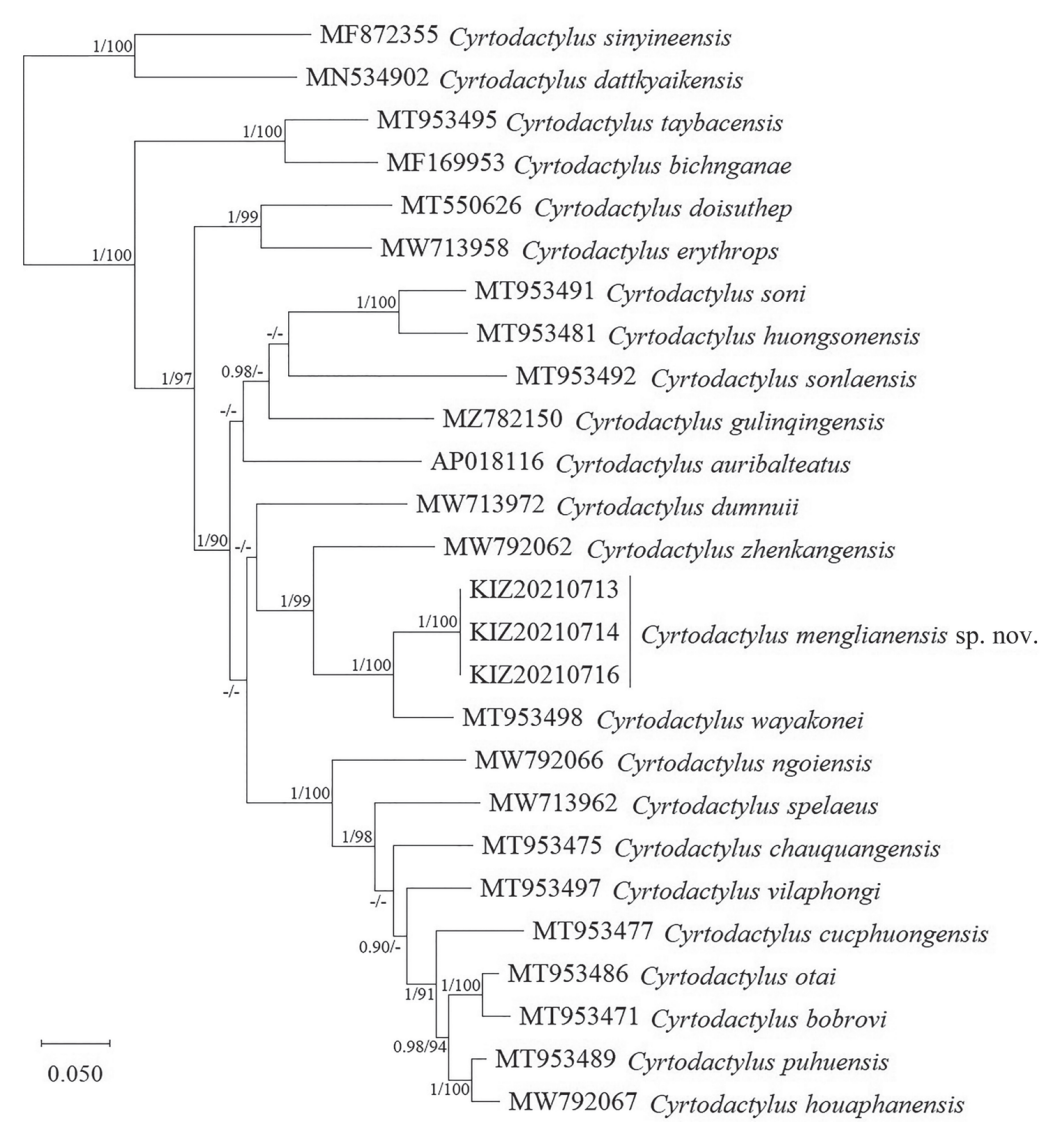
Bayesian phylogram of the *Cyrtodactyluschauquangensis* species group inferred from the ND2 gene and its flanking tRNAs. Numbers before slashes indicate Bayesian posterior probabilities and numbers after slashes indicate the ML ultrafast bootstrap. The symbol “–” represents the value below 0.90/90.

The obtained sequence alignment is 1397 bp in length. The sequences of the three specimens collected from Menglian County, Yunnan, China, were nested within the *Cyrtodactyluschauquangensis* species group and represented a distinct clade sister to *C.wayakonei* Nguyen, Kingsada, Rösler, Auer & Ziegler, 2010 with strong support (Fig. 1). The interspeciﬁc pairwise distances between the newly collected specimens and other members of *C.chauquangensis* species group ranged from 7.2% to 18.4% (Table 1).

**Table 1.** Mean uncorrected pairwise genetic distances (%) among the species of the *Cyrtodactyluschauquangensis* species group and outgroups based on the ND2 gene and its flanking tRNAs.

	1	2	3	4	5	6	7	8	9	10	11	12	13	14	15	16	17	18	19	20	21	22	23
1 *Cyrtodactylusmenglianensis* sp. nov.																							
2 *Cyrtodactylusauribalteatus*	12.2																						
3 *Cyrtodactylusbichnganae*	18.4	18.3																					
4 *Cyrtodactylusbobrovi*	15.0	13.9	19.7																				
5 *Cyrtodactyluschauquangensis*	12.6	13.3	18.1	8.6																			
6 *Cyrtodactyluscucphuongensis*	15.0	14.5	19.9	7.9	8.4																		
7 *Cyrtodactylusdoisuthep*	15.5	13.6	16.6	15.9	14.4	15.9																	
8 *Cyrtodactylusdumnuii*	11.4	11.9	17.0	13.7	12.3	14.4	14.3																
9 *Cyrtodactyluserythrops*	13.9	13.8	16.7	14.8	13.5	14.7	11.0	13.4															
10 *Cyrtodactylusgulinqingensis*	14.3	13.2	18.1	13.8	14.0	14.0	14.1	12.9	13.8														
11 *Cyrtodactylushouaphanensis*	14.8	14.6	19.4	6.5	9.0	7.5	15.5	14.2	14.9	14.1													
12 *Cyrtodactylushuongsonensis*	14.3	13.4	17.7	14.3	12.5	14.3	14.7	13.9	14.3	12.4	14.7												
13 *Cyrtodactylusngoiensis*	13.1	13.2	18.2	11.1	10.5	10.7	14.9	12.0	14.2	13.1	11.3	13.1											
14 *Cyrtodactylusotai*	15.2	14.6	19.1	3.6	9.1	8.4	16.3	15.6	16.4	15.6	6.8	14.7	12.2										
15 *Cyrtodactyluspuhuensis*	14.2	13.4	18.9	5.7	8.0	7.1	14.7	12.9	14.2	13.6	2.8	13.8	10.5	6.2									
16 *Cyrtodactylussoni*	13.4	13.0	18.2	14.3	13.6	14.4	14.2	13.1	14.2	13.0	15.3	6.7	14.0	14.7	14.2								
17 *Cyrtodactylussonlaensis*	17.2	16.2	19.4	17.5	16.8	18.1	16.2	16.8	17.3	14.8	18.0	15.0	16.2	17.7	18.0	15.2							
18 *Cyrtodactylusspelaeus*	14.6	13.9	18.3	10.0	9.2	10.4	15.7	13.4	15.0	13.9	10.4	14.3	11.1	11.3	9.1	14.3	17.7						
19 *Cyrtodactylustaybacensis*	16.8	15.6	9.3	17.1	15.5	17.3	15.7	14.9	16.4	16.3	17.3	16.1	16.6	18.3	16.7	15.6	18.9	16.5					
20 *Cyrtodactylusvilaphongi*	14.1	13.4	17.8	8.1	7.3	8.2	14.2	13.3	14.0	13.5	8.2	14.2	9.5	9.1	7.0	13.5	16.9	9.6	16.5				
21 *Cyrtodactyluswayakonei*	7.2	13.1	18.0	15.5	13.1	15.5	16.3	12.7	15.6	15.3	14.7	15.1	12.2	15.4	14.2	13.9	16.4	15.2	17.5	13.7			
22 *Cyrtodactyluszhenkangensis*	10.7	12.0	18.4	14.1	13.2	13.8	15.5	11.8	14.0	12.9	13.9	13.4	13.2	15.5	13.2	13.7	17.3	14.0	15.8	13.6	11.9		
23 *Cyrtodactylusdattkyaikensis*	18.3	17.2	21.4	18.8	18.0	19.4	18.0	17.0	16.8	16.8	19.9	17.2	18.0	21.5	18.8	17.9	21.8	19.3	18.4	17.8	19.9	17.9	
24 *Cyrtodactylussinyineensis*	18.8	17.8	18.9	18.7	17.5	18.1	18.2	17.0	18.7	18.9	18.9	19.1	17.5	20.9	18.1	18.7	21.9	18.9	17.9	18.1	19.4	18.4	13.1

### ﻿ Cyrtodactylus menglianensis sp. nov.

Taxon classificationAnimaliaSquamataGekkonidae

00DE1CD0-CAA8-5D1F-B7F4-F40310046C8E

http://zoobank.org/6E8C0453-145B-4862-9A87-BC3F4F4344FD

**Figure 2.**
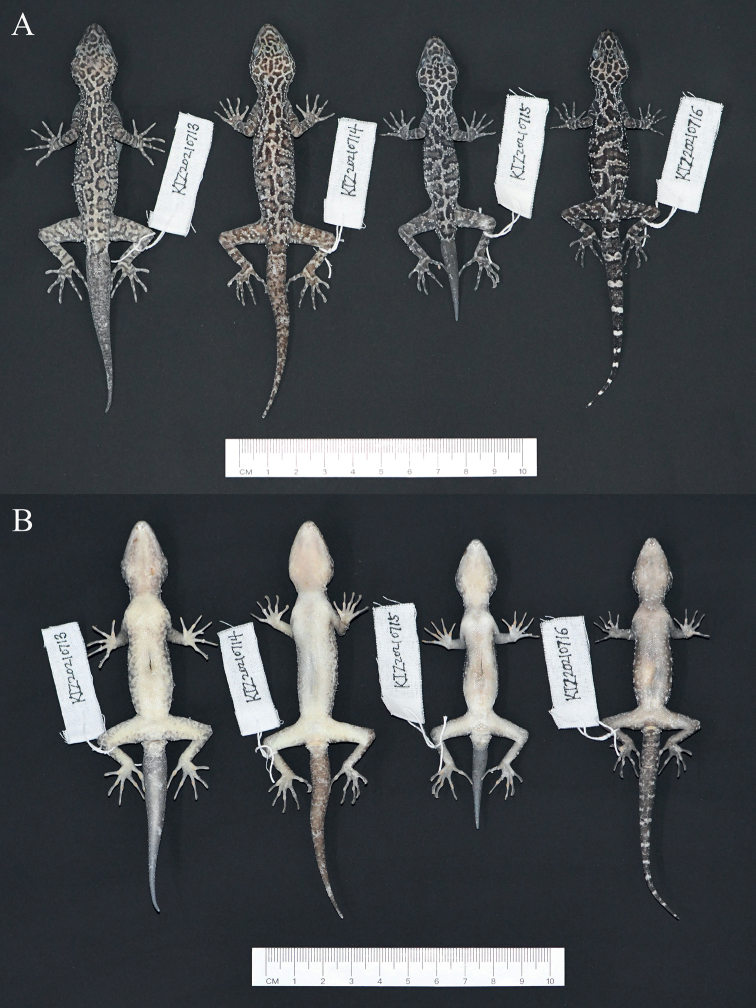
Type series of *Cyrtodactylusmenglianensis* sp. nov. in preservative **A** dorsal views **B** ventral views.

**Figure 3.**
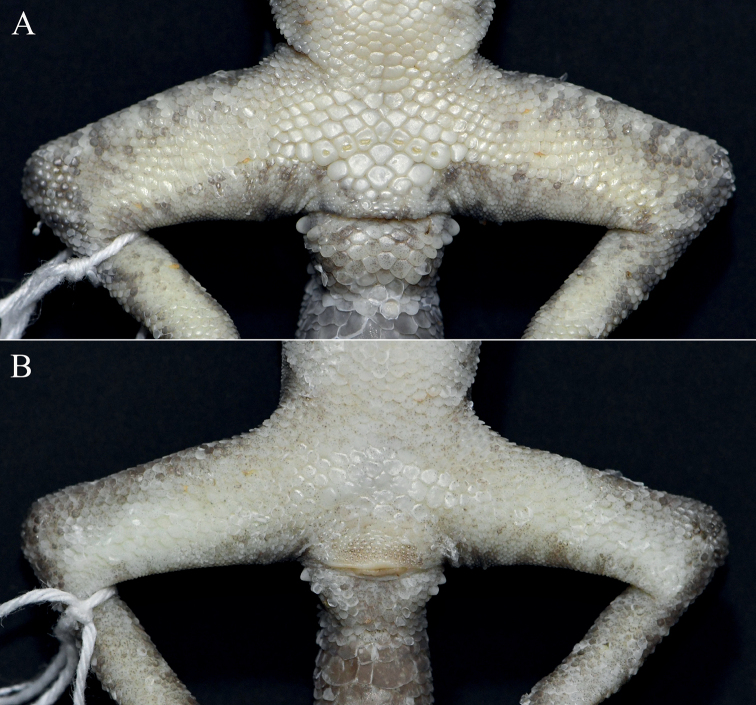
Close-up views of the femoral and precloacal regions of *Cyrtodactylusmenglianensis* sp. nov. **A** male holotype (KIZ20210713) **B** female paratype (KIZ20210714).

**Figure 4.**
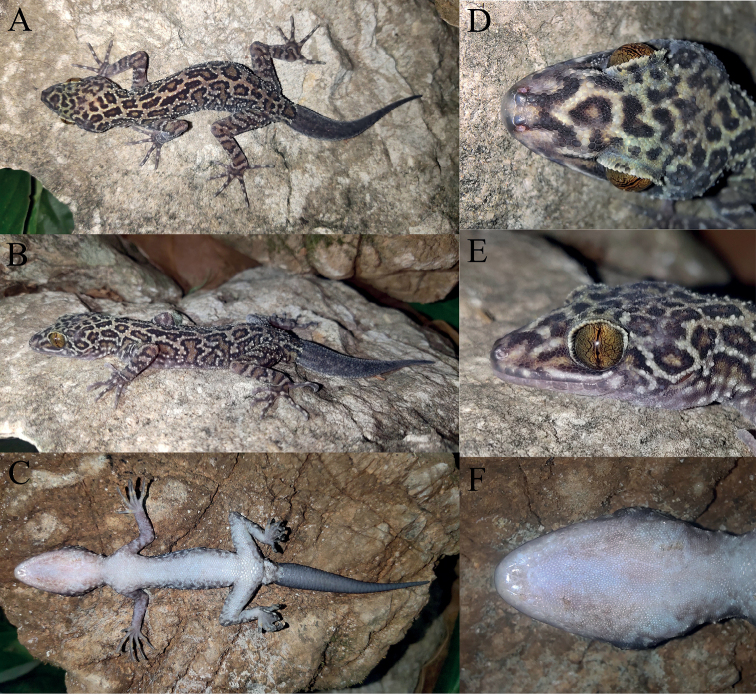
Holotype (KIZ20210713) of *Cyrtodactylusmenglianensis* sp. nov. in life **A** dorsal view of whole body **B** lateral view of whole body **C** ventral view of whole body **D** dorsal view of head **E** lateral view of head **F** ventral view of head.

**Figure 5.**
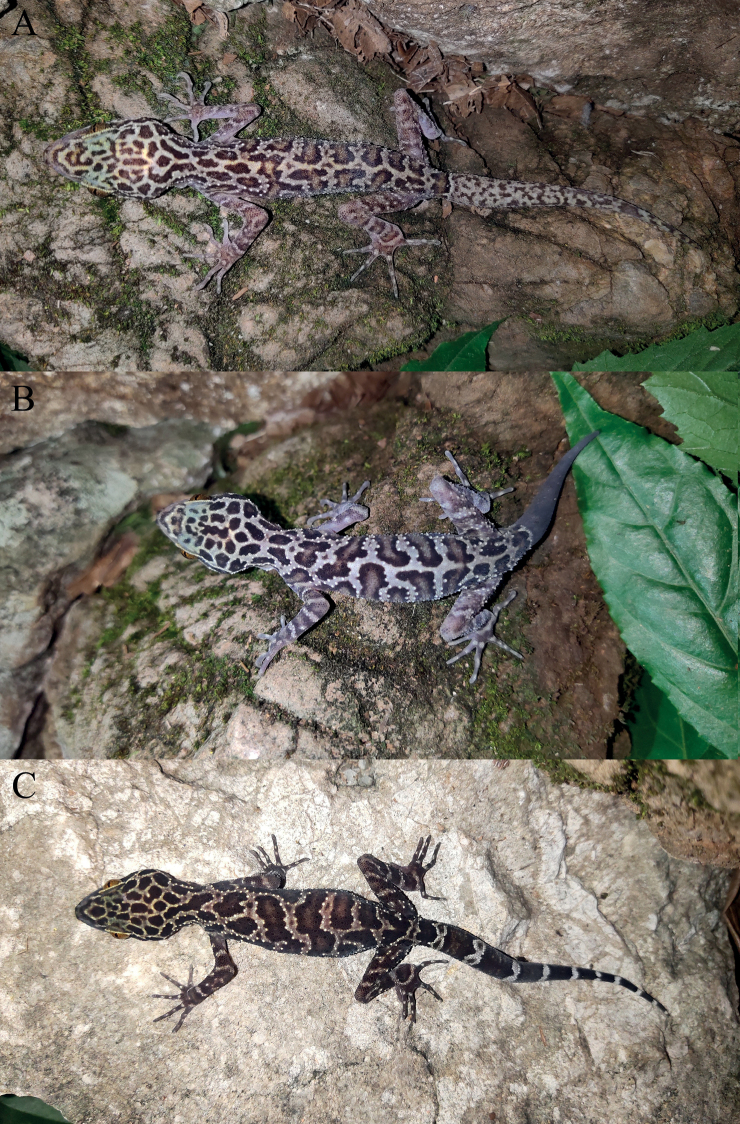
Paratypes of *Cyrtodactylusmenglianensis* sp. nov. in life **A** female paratype (KIZ20210714) **B** male paratype (KIZ20210715) **C** female paratype (KIZ20210716).

Figures 2 , 3 , 4 , 5

#### Type material.

***Holotype*.** KIZ20210713, adult male, collected on 18 July 2021 by Shuo Liu from Menglian County, Puer City, Yunnan Province, China (22°20'11"N, 99°34'29"E, 980 m elevation).

***Paratypes*.** KIZ20210714 and KIZ20210716, two adult females; KIZ20210715, adult male; all collected on 19 July 2021 by Shuo Liu from the same locality as the holotype.

#### Etymology.

The specific epithet refers to Menglian County, the locality where the new species was found. We propose “Menglian Bent-toed Gecko” for the common English name and “孟连裸趾虎” (Mèng Lián Luǒ Zhǐ Hǔ) for the common Chinese name of the new species.

#### Diagnosis.

Medium body size (SVL 67.7–78.1 mm in adults); ventrolateral folds present with interspersed small tubercles; seven precloacal pores in a continuous series in males, absent in females; femoral scales not enlarged; femoral pores absent; two postcloacal tubercles on each side; 17–22 lamellae under finger IV, 21–23 lamellae under toe IV; one or two rows of subcaudals enlarged; dark postocular streak and nuchal loop absent; six or seven dark irregular dorsal bands between limb insertions, most bands discontinuous.

#### Description of holotype.

Adult male, SVL 77.8 mm; head distinguished from neck, moderately long (HL/SVL 0.28), relatively widened (HW/HL 0.76), slightly depressed (HH/HL 0.45); two supranasals separated by one internasal; nares oval, surrounded by supranasal, rostral, first supralabial, and three postnasals; loreal region concave; snout long (SE/HL 0.43), round anteriorly, longer than diameter of orbit (OD/SE 0.63); snout scales small, round, granular, larger than those in frontal and parietal regions; eye large (OD/HL 0.27), pupils vertical; upper eyelid fringe with spinous scales; ear opening oval, small (ED/HL 0.09); rostral wider than high (RH/RW 0.58), medially divided dorsally by a suture, reaching to approximately half down rostral, in contact with first supralabial and nostrils laterally, and supranasals and internasal dorsally; mental triangular, narrower than rostral (MW/RW 0.82), slightly wider than high (ML/MW 0.94); two postmentals, enlarged, in contact posteriorly, bordered by mental anteromedially, first infralabial anterolaterally, two enlarged chin scales posterolaterally, and small chin scales posteriorly; 10/12 supralabials; 9/9 infralabials.

Body slender (AG/SVL 0.39), ventrolateral folds slightly developed with interspersed small tubercles; dorsal scales granular; dorsal tubercles round and weakly keeled, four or five times larger than the size of adjoining scales, conical, present on neck, back and tail base, each surrounded by 10 granular scales, in 19 irregular longitudinal rows at the midbody, 26 paravertebral tubercles; gular region with homogenous smooth scales; ventral scales smooth, larger than those of dorsum, round, subimbricate, largest posteriorly, in 29 longitudinal rows at midbody; precloacal groove absent; a patch of precloacal scales significantly enlarged; seven precloacal pores in a continuous series, the two on the edge round, the one in the middle pitted, others horizontally elongated; femoral scales not enlarged; femoral pore absent.

Fore and hind limbs moderately slender (ForeaL/SVL 0.17, SL/SVL 0.20); dorsal surface of forelimbs covered by a few weakly developed tubercles; interdigital webbing absent; lamellae under finger IV 22/21, under toe IV 23/22; relative length of fingers I < II < V < III < IV, relative length of toes I < II < III < V < IV.

Tail regenerated (TaL 60.8 mm); 2/2 postcloacal tubercles; dorsal tail base with tubercles; subcaudals smooth, enlarged but arranged irregularly.

#### Color of holotype in life.

Dorsal ground color brownish yellow; dorsal surface of head with irregular brown blotches with black edges, largest at occiput; nuchal loop absent; dorsum with many irregular brownish black blotches with black edges, forming eight transverse discontinuous bands faintly, one on the neck, one between hind limbs, and six between fore and hind limb insertions; dorsal surfaces of limbs with brown bands with black edges; a brown band with black edge on dorsal tail base, dorsal surface of regenerated tail greyish black; ventral surface of head, limbs, and body greyish white; ventral surface of regenerated tail grey; iris bronze.

#### Color of holotype in preservative.

The color pattern very much resembles that in life. Brownish yellow dorsal ground color turned to greyish white, the brown blotches and bands with black edges remained; ventral surface faded to pale white; iris became white.

#### Variations.

The paratypes resemble the holotype except that the female KIZ20210714 has a longer regenerated tail, and the female KIZ20210716 has a longer original tail with one or two rows of subcaudals enlarged, and they both have no precloacal pores; the male KIZ20210715 has a smaller body size and much shorter regenerated tail; other morphometric and meristic differences are presented in Table 2. Color patterns of the paratypes also resemble the holotype except that the dark bands on the dorsum are relatively more distinct, and there are 10 black and white rings on the original tail of the female KIZ20210716.

**Table 2.** Measurements (mm) and meristic data for the type series of *Cyrtodactylusmenglianensis* sp. nov. Abbreviations defined in Materials and methods. “^*^” represents regenerated tail and “^#^” represents original tail.

	KIZ20210713 Holotype	KIZ20210714 Paratype	KIZ20210715 Paratype	KIZ20210716 Paratype
	Male	Female	Male	Female
SVL	77.8	78.1	69.1	67.7
TaL	60.8^*^	62.9^*^	33.2^*^	70.6^#^
HH	9.7	9.7	8.3	8.7
HL	21.7	21.8	19.4	19.2
HW	16.4	15.8	14.8	14.5
OD	5.9	6.9	5.1	5.3
SE	9.3	9.2	8.2	8.3
EE	6.3	5.9	5.6	5.6
IND	3.1	3.1	2.9	2.6
IOD	3.3	3.5	2.8	2.7
ED	1.9	1.3	1.3	1.2
AG	30.5	34.6	27.8	28.4
ForeaL	13.0	12.9	11.4	10.6
SL	15.7	15.6	14.0	13.1
RW	3.8	3.4	2.9	3.0
RH	2.2	2.1	1.9	1.8
MW	3.1	3.2	2.9	3.8
ML	2.9	2.6	2.6	2.0
SPL	10/12	9/10	8/9	10/9
IFL	9/9	9/9	9/8	9/7
I	1	1	1	1
PM	2	2	2	2
GSDT	10	10	10	10
DTR	19	21	20	18
PVT	26	27	25	29
V	29	26	26	28
EFS	0	0	0	0
PP	7	0	7	0
FP	0	0	0	0
PAT	2/2	2/2	2/2	2/2
LF4	22/21	19/18	18/17	21/22
LT4	23/22	22/22	22/21	23/23

#### Distribution.

**Figure 6.**
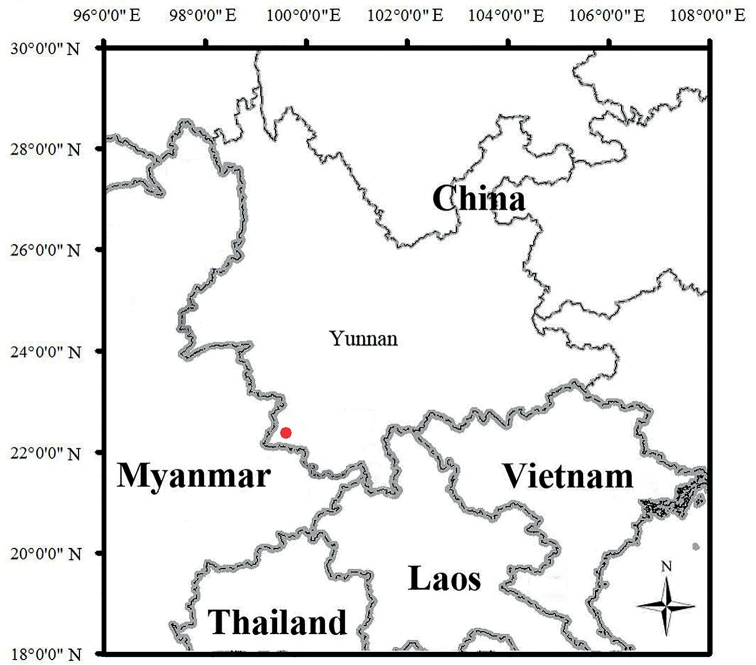
Map showing the type locality (red dot) of *Cyrtodactylusmenglianensis* sp. nov. in Menglian County, Puer City, Yunnan Province, China.

The new species is currently known only from the type locality (Fig. 6) in Menglian County, Puer City, Yunnan Province, China.

#### Natural history.

All specimens were collected at night on large stones or cliffs of the karst formations in a park. The surrounding habitats was karst forest, there is a plank road and a river nearby.

#### Comparisons.

*Cyrtodactylusmenglianensis* sp. nov. is distinguishable from all other members of the *C.chauquangensis* species group by a unique combination of morphological characters. *Cyrtodactylusmenglianensis* sp. nov. differs from *C.auribalteatus* Sumontha, Panitvong & Deein, 2010; *C.bichnganae* Ngo & Grismer, 2010; *C.doisuthep* Kunya, Panmongkol, Pauwels, Sumontha, Meewasana, Bunkhwamdi & Dangsri, 2014; *C.dumnuii* Bauer, Kunya, Sumontha, Niyomwan, Pauwels, Chanhome & Kunya, 2010; *C.erythrops* Bauer, Kunya, Sumontha, Niyomwan, Panitvong, Pauwels, Chanhome & Kunya, 2009; *C.gulinqingensis* Liu, Li, Hou, Orlov & Ananjeva, 2021; *C.hekouensis* Zhang, Liu, Bernstein, Wang & Yuan, 2021; *C.huongsonensis* Luu, Nguyen, Do & Ziegler, 2011; *C.ngoiensis* Schneider, Luu, Sitthivong, Teynié, Le, Nguyen & Ziegler, 2020; *C.soni* Le, Nguyen, Le & Ziegler, 2016; *C.sonlaensis* Nguyen, Pham, Ziegler, Ngo & Le, 2017; and *C.zhenkangensis* Liu & Rao, 2021 by not having enlarged femoral scales and femoral pores (vs having enlarged femoral scales and femoral pores).

*Cyrtodactylusmenglianensis* sp. nov. differs from *C.puhuensis* Nguyen, Yang, Le, Nguyen, Orlov, Hoang, Nguyen, Jin, Rao, Hoang, Che, Murphy & Zhang, 2014 and *C.taybacensis* Pham, Le, Ngo, Ziegler & Nguyen, 2019 by not having enlarged femoral scales (vs having enlarged femoral scales). In addition, *C.menglianensis* sp. nov. differs from *C.puhuensis* by having more precloacal pores in males (seven vs five) and differs from *C.taybacensis* by having fewer precloacal pores in males (seven vs 11–13).

*Cyrtodactylusmenglianensis* sp. nov. differs from *C.cucphuongensis* Ngo & Chan, 2011 by having precloacal pores in males (vs not having precloacal pores in males).

*Cyrtodactylusmenglianensis* sp. nov. differs from *C.bobrovi* Nguyen, Le, Pham, Ngo, Hoang, Pham & Ziegler, 2015; *C.chauquangensis* Hoang, Orlov, Ananjeva, Johns, Hoang & Dau, 2007; *C.houaphanensis* Schneider, Luu, Sitthivong, Teynié, Le, Nguyen & Ziegler, 2020; *C.otai* Nguyen, Le, Pham, Ngo, Hoang, Pham & Ziegler, 2015; *C.spelaeus* Nazarov, Poyarkov, Orlov, Nguyen, Milto, Martynov, Konstantinov & Chulisov, 2014; and *C.vilaphongi* Schneider, Nguyen, Le, Nophaseud, Bonkowski & Ziegler, 2014 by not having dark postocular streak and nuchal loop (vs having very obvious dark postocular streak and not obvious nuchal loop).

*Cyrtodactylusmenglianensis* sp. nov. differs from *C.martini* Ngo, 2011 by not having enlarged femoral scales (vs having indistinctly enlarged femoral scales), having fewer longitudinal ventral scale rows (26–29 vs 39–43), having more precloacal pores in males (seven vs four), and having more white rings on the original tail (10 vs 7).

*Cyrtodactylusmenglianensis* sp. nov. differs from *C.wayakonei* Nguyen, Kingsada, Rösler, Auer & Ziegler, 2010 by having fewer longitudinal ventral scale rows (26–29 vs 31–35), not having precloacal pores in females (vs having precloacal pores in females), and having more white rings on the original tail (10 vs 6).

## ﻿Discussion

**Figure 7.**
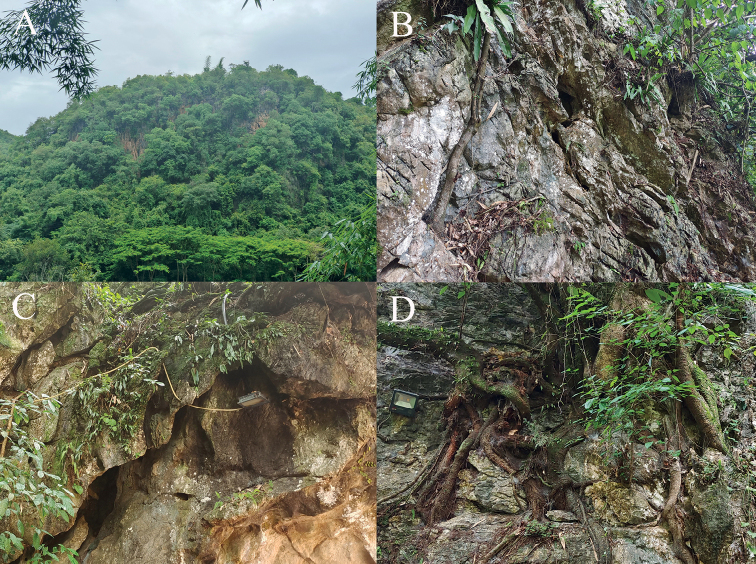
Habitat of *Cyrtodactylusmenglianensis* sp. nov. at the type locality **A** distant view **B** close view **C, D** lamps on the limestone cliffs.

The new species was found in a park just beside the county seat. There is a plank road along the limestone cliffs in the park, and there are many lamps on the limestone cliffs along the plank road (Fig. 7). These lamps light up every night and have some influence on nocturnal animals. We found that the populations of nocturnal animals there were very small, including that of the new species. Next to the park is a small nature reserve, which focuses only on the protection of several rare plants. We suggest that this nature reserve also include animals and the karst formations into their protection.

There are still many karst landforms in southern Yunnan which have not been surveyed in detail. Additional cryptic new species of *Cyrtodactylus* are likely to be found in these areas. It is necessary to strengthen the protection of these karst landforms and to survey these areas.

## Supplementary Material

XML Treatment for Cyrtodactylus menglianensis
